# Engaging Rural High School Youth in E-cigarette Prevention and Advocacy

**Published:** 2021-08

**Authors:** Melinda J. Ickes, Olivia Zidzik, Nathan L. Vanderford

**Affiliations:** University of Kentucky; University of Kentucky; University of Kentucky

## Introduction

Tobacco is still the leading cause of preventable disease and death in the United States, and behaviors such as cigarette smoking and using tobacco products, including e-cigarettes, often begin during the formative life stages of youth and adolescence.^1^ The state of Kentucky is particularly burdened by the tobacco epidemic, with one of the nation’s highest smoking prevalence and lung cancer incidence.^2^ While the majority of prevention and education programming has focused on deterring cigarette use in general, youth in Kentucky reported higher usage rates of other tobacco products, including e-cigarettes, when compared to their national-level counterparts.^2^ Recent national data reinforces that over 3.6 million young people across the United States are currently using e-cigarettes.^3^ In Kentucky, e-cigarette use among 10^th^ grade students increased 200% statewide over the last two years,^4^ which emphasizes the magnitude of the problem.

Public health professionals, including the Surgeon General, continue to express concern as research emerges on the short- and potentially long-term health consequences of youth e-cigarette use.^1^ E-cigarette use targets every area of a young person’s growing body, including but not limited to: lungs (bronchiolitis obliterans, e-cigarette or vaping product use-associated lung injury, etc.); brain (difficulty concentrating, irritability, etc.); and the cardiovascular system (increased heart rate, blood pressure, etc.).^1,5–8^ Despite the potential threats that e-cigarettes pose, young people report reduced perceived harm and still choose to use.^9^

While the data and risks of use are alarming, they underscore the importance of tailored health promotion strategies, including prevention education and opportunities to engage youth in prevention efforts. Fundamentally, there is a lack of e-cigarette education programs,^1,10^ which may relate to the perceived reduced risk of using e-cigarettes among youth. This presents a need for innovative programs that empower young people to take a stance in changing the e-cigarette epidemic in their own schools and communities.^11^ When youth understand that they are being manipulated by tobacco companies, they are persuaded to get involved in tobacco control,^11^ and this might also support e-cigarette prevention and advocacy efforts. Youth in Appalachia demonstrate desire to influence tobacco use and policy to improve health equity.^12^ Similarly, young people in Generation Z believe they have the power to transform the world for the better with the majority wanting to learn what they can do to make a difference.^13^ Providing young people with opportunities to make a difference through ongoing training and support in tobacco prevention has promise.^12^ Connecting these efforts with the e-cigarette epidemic through a peer-to-peer model could be an innovative way to combine best practices for tobacco prevention, youth engagement, and combat the challenge currently faced with the surge in e-cigarette use among young people.

The purpose of this study was to pilot a peer led e-cigarette prevention and advocacy training and determine attitudes and self-efficacy among participating rural high school leaders post-training. Information will provide valuable insight in engaging youth in future efforts to combat the e-cigarette epidemic.

## Methods

### Study Design & Procedures

The study incorporated a one-group, post-test design, recruiting participants from an existing student leadership group (*N* = 16) who participated in an e-cigarette prevention and empowerment training in December 2019. Approval was obtained from the Institutional Review Board of the authors’ institution. Participants were sent a link to the online survey through Qualtrics, a secure survey database, at 4-months post-training.

### Sample

Students already engaged in the Appalachian Career Training in Oncology (ACTION) Program at the University of Kentucky participated in an e-cigarette prevention and empowerment training. The ACTION program provides advanced cancer education and training to high school students who are from Appalachian Kentucky as a means of preparing them to pursue cancer-related careers. Students engage in a variety of research, clinical, education, and outreach activities for at least 10 hours/week during the academic year and for 40 hours/week during the summer. ACTION program participants at the time this intervention occurred were 71.2% female, 28.8% male, 40.7% low income, 31.4% first generation college students, and 9.2% members of an underrepresented minority. A total of 16 participants completed the post-survey and were included in the study/analysis described within this paper.

### Training Description

#iCANendthetrend is a peer-led e-cigarette prevention and empowerment program developed by the University of Kentucky researchers and college students engaged in tobacco prevention. The program aims to educate elementary, middle, and high school students on the dangers of e-cigarette use among youth while empowering youth to make a difference in their local communities through a peer-to-peer approach. The tailored program covers the health consequences, social repercussions, and financial costs of nicotine dependence in alignment with the National Health Education Standards.^14^ In addition, college facilitators inform youth about the ways they are targeted by the tobacco/e-cigarette industry and equip them with refusal skills and quitting tools through interactive approaches. Since it was established in February 2019, the program has reached over 5,000 youth across Kentucky.

#iCANendthetrend facilitators partnered with the ACTION group to implement a tailored training, “A Leader’s Guide to E-cigarette Advocacy,” combining general e-cigarette awareness topics as mentioned above with additional hands-on training in e-cigarette community and policy advocacy. Student leaders worked jointly during two 50-minute sessions to explore reasons for increased use among their generation and specifically how they are being targeted by the industry through flavors, marketing, and social norm strategies. In addition, participants engaged in power mapping in order to assess how they can become better advocates to fight against the youth e-cigarette epidemic in their own community. Students were also given resources to support planning action-oriented e-cigarette prevention events that they could host at their school or in their local communities. Advocacy curriculum was tailored based on previous successful intervention approaches in tobacco policy advocacy in similar communities.^12^

### Measures

As part of the impact evaluation, a 12-item measure was developed to assess the students’ risk perceptions, attitudes, and empowerment/self-efficacy.^15–17^ Risk perception items assessed individuals’ belief in harms associated with e-cigarette use, with a 5-point Likert-scale ranging from strongly disagree to strongly agree. A sample item is, “Youth are at risk for nicotine addiction if they use e-cigarettes.” E-cigarette attitude items assessed individuals’ general attitudes toward e-cigarettes (e.g., personal concern) as well as strategies toward e-cigarette prevention (e.g., education, policy), with a 5-point Likert-scale ranging from strongly disagree to strongly agree. A sample item is, “I support a policy that does not allow e-cigarette use inside and outside on school property.” Finally, empowerment and self-efficacy items assessed individuals’ belief in their ability to influence behaviors and select effective prevention strategies, with a 5-point Likert-scale ranging from strongly disagree to strongly agree. A sample item is, “There is something I can do to change the trend of using e-cigarette or vaping products in my school.”

#### Data analysis

The Likert categories were collapsed into agree and disagree levels and the “agree” responses are summarized herein. Frequency distributions were used to summarize outcomes.

## Results

Participating students (n=16) were either 16 (50%) or 17 (50%) years of age, with the majority in the 11th grade (75%), female (56.25%), and White (81.25%). These demographics generally align with those participating in the ACTION program (Table 1).

### Risk Perceptions & Attitudes

Students were asked several Likert scale questions to assess their risk perceptions and attitudes toward e-cigarettes post-training (Table 2). All participants agreed that youth are at risk for nicotine addiction and that e-cigarette aerosol is dangerous to breathe. Students all also expressed concern regarding e-cigarette use among people their age and believed their age group is being targeted by companies to buy these products. When specifically asked about attitudes toward e-cigarette prevention, 93.8% agreed that education regarding the dangers of tobacco use is an important strategy. All supported tobacco control policies in their community to prohibit e-cigarette use in indoor workplaces and public spaces as well as on school property. Finally, the majority of participants (93.8%) agreed that e-cigarettes should be regulated by the federal government.

### Empowerment/Self-efficacy

Students were also asked several Likert scale questions to determine their sense of empowerment and self-efficacy in participating in prevention and/or advocacy efforts post-training (Table 3). At the individual level, the far majority (87.5%) agreed that they would be comfortable encouraging a friend to quit vaping. Similarly, 87.5% agreed there was something they could do to change the trend of using e-cigarettes in their school and they could select effective strategies to educate young people to lower tobacco use in their community. In addition, 87.5% expressed interest in forming a student group to support efforts to lower tobacco use in their community.

## Discussion

The #iCANendthetrend *“A Leader’s Guide to E-cigarette Advocacy”* training assessed rural student leaders’ attitudes toward e-cigarettes and e-cigarette prevention, risk perceptions of e-cigarettes, and how empowered one became to make a change in one’s state or local communities as a result of the training. Although exploratory in nature, we believe our study is the first of its kind to train youth from rural, Appalachian communities specifically in e-cigarette prevention and advocacy. Findings reinforce numerous positive outcomes of this peer-led training among participating youth, including an awareness of evidence-based information on the risks of e-cigarette use among young people and the desire of young people to impact the e-cigarette epidemic in their communities. Given the lack of research in this area, the findings are promising and help provide a framework for future training and community engagement opportunities with youth from Appalachian and rural communities. Youth in Appalachian communities typically describe a deeply entrenched tobacco culture, reinforced by social and environmental factors,^18^ which is a common barrier to engaging in tobacco prevention and advocacy efforts. However, the e-cigarette epidemic among young people may be an opportunity to address these barriers given the risk of use among youth across all communities.

Students all expressed concern regarding e-cigarette use among people their age. Given the surge in e-cigarette use among young people nationally, as well as in Kentucky,^3,4^ this is not surprising. However, it does reinforce that other young people care and are concerned that people their age are using these products, which can support peer-led prevention efforts. Given decreased risk perceptions of e-cigarettes expressed by many youth,^9^ students were introduced to some of the emerging consequences of e-cigarette use, specifically those relevant to young people. It is noteworthy that post-training, youth recognized the potential consequences of youth e-cigarette use including risk of nicotine addiction and that e-cigarette aerosol is dangerous to breathe. Oftentimes, talking about the long-term health consequences of tobacco use does not resonate with young people.^19^ Reinforcing how use of these products can impact them and others their age aligns with developmentally appropriate health standards and also provides an opportunity for peers to approach the topic in a very relatable manner.

Similarly, post-training, all participating youth believed that their age group is being targeted by companies to buy e-cigarette products. The relationships between the marketing activities and manipulation strategies used by the tobacco companies and the use of tobacco, including use among young people, have become clear over decades of research.^20^ Tobacco and related industries have used appealing flavors, deceptive product designs, misleading unscientific marketing of ‘reduced harm’ products, influencer and brand-sponsorships, strategic product placement, and easy access, to name a few targeted strategies.^20^ While research continues to emerge specific to e-cigarettes, similar tactics are being used and influencing youth use.^21,22^ As youth gain awareness of these manipulating strategies, they tend to be more interested in engaging in prevention and advocacy efforts.^11^

While many tobacco prevention approaches tend to focus on individual-level education such as health risks, there is a need to consider the multi-level influences within the best practices model.^11,19^ Students participating in the training were introduced to the larger tobacco control landscape, including relevant policies which support decreased tobacco use^19^ among youth and in the surrounding communities. Post-training, students supported tobacco control policies in their community to prohibit e-cigarette use in indoor workplaces and public spaces as well as on school property. Similarly, the majority of participants also agreed that e-cigarettes should be regulated by the federal government. Little is known regarding youth perceptions toward tobacco policies specific to e-cigarettes, so this is an area warranting additional exploration. Given the current gaps in the regulation of e-cigarettes and the lack of consistency in local and statewide policies,^23^ young people may provide impetus to strengthen existing policies given they recognize the impact policies can have on e-cigarette use among other youth. Students who have more positive attitudes toward tobacco policies are more likely to participate in advocacy efforts,^24^ reinforcing why this topic should be integrated into all youth tobacco prevention training and outreach opportunities. Such integration supports Best Practices in Tobacco Control,^19^ and may strengthen tobacco prevention and youth advocacy efforts toward evidence-based tobacco policies in communities with weaker policies, particularly those not including e-cigarettes.

Another goal of the training was to empower young people and enhance their self-efficacy in making a difference in their communities. Most students agreed there was something they could do to change the trend of using e-cigarettes in their school, and they could select effective strategies to educate young people to lower tobacco use in their community. While peer approaches have shown to be effective with tobacco prevention in general,^25^ no peer-led approaches targeting e-cigarette prevention have been published to date. Given the youth e-cigarette epidemic, there is promise in exploring this as an avenue to promote prevention efforts to support tobacco prevention best practices.^19^ Almost all participating youth also agreed that they would be comfortable encouraging a friend to quit vaping post-training, so providing support for young people to do so is imperative. New youth-friendly treatment resources have emerged and shown success,^26^ thus reinforcing this message to other young people might encourage them to share these resources with other people their age. Additional research is needed to not only understand youth facilitators and barriers to quitting e-cigarettes, but also how other youth can support people their age to be successful in their quitting efforts.

Finally, 87.5% of participating youth expressed interest in forming a student group to lower tobacco use in their community. Future efforts are needed to support youth engagement in this area.^12^ While youth should be encouraged to engage in these efforts, it is important to recognize the need to tie to best practices^19^ and for youth to feel supported to maximize the impact and sustainability efforts.^15^ Building training and long-term engagement opportunities into existing student groups, such as the ACTION group with whom this training program partnered, may be a good platform to begin school- and community-based prevention and advocacy efforts.

### Strengths and Limitations

Strengths of this study include being the first of its kind to report on a peer-led e-cigarette prevention and advocacy training opportunity. The inclusion of students from rural, Appalachian communities who were participating in a leadership initiative provided a captive audience of student leaders and those interested in this topic. While findings of this study provide a foundation for future research and youth engagement opportunities in e-cigarette prevention and advocacy, the study had several limitations that must be considered. While a pre-test, post-test design would have been ideal, due to data collection constraints of students not completing the baseline prior to the training, we were unable to collect true baseline data to make pre- to post-comparisons. Given all participants were students in the ACTION program, they may have had an enhanced interest in this topic prior to completing the training. However, anecdotally, our peer facilitators were surprised at the lack of knowledge specific to the health consequences and manipulation by the industry during the training session. Future studies would ideally include a larger sample, a broader range of high school students representing diverse communities, as well as a control group to enhance generalizability of the findings.

### Implications for Practice

The youth e-cigarette epidemic continues to be a concerning public health issue. Unfortunately, those who live in rural, Appalachian communities with greater tobacco use and related health disparities are not immune to youth e-cigarette use. Youth engagement in tobacco prevention efforts is an effective strategy to bring about individual and community-level changes. Youth participating in this study were concerned about their peers using e-cigarettes and also recognized the opportunity to make a difference. The peer-led approach used in this study provides an opportunity for young people to connect with others who are facing this issue and to see them as role models who are striving to make a difference.

However, continued support and opportunities for engagement are necessary to maximize evidence-based and sustainable efforts involving youth. Lessons learned with this formative research can help inform long-term programming and engagement opportunities that build on the success of peer-led approaches in tobacco prevention to impact the youth e-cigarette epidemic.

## Figures and Tables

**Table 1.** Participant demographics.

Demographic	N	%
***Age***		
16	8	50.00
17	8	50.00

***Sex***		
Female	9	56.25
Male	7	43.75

***Race***		
Asian	1	6.25
Black	0	0
More than one race	2	12.50
White	13	81.25
Other	0	0

***Ethnicity***		
Hispanic or Latino	0	0
Not Hispanic or Latino	16	100.00

***Grade***		
10	4	25.00
11	12	75.00

**Table 2.** Risk Perceptions and Attitudes Toward E-cigarettes Post-Training

Variable	Post-training % Agreed (n)
**Risk Perceptions of E-cigarettes**	
Youth are at risk for nicotine addiction if they use e-cigarettes.	100 (16)
E-cigarette aerosol is dangerous to breathe.	100 (16)

**E-cigarette Attitudes**	
Consider the following statements and indicate how strongly you agree or disagree. I am concerned about e-cigarette use among people my age.	100 (16)
Companies target people my age to use and buy e-cigarette or vaping products.	100 (16)

**Attitudes Toward E-cigarette Prevention**	
In order to lower tobacco use, educating people about the dangers of tobacco use is an important strategy.	93.75 (15)
I support a policy in my community that does not allow e-cigarette use (e.g. Juul and vaping) in indoor workplaces and public places.	100 (16)
I support a policy that does not allow e-cigarette use (e.g. Juul and vaping)inside and outside on school property.	100 (16)
E-cigarettes should be regulated by the federal government.	93.75 (15)

**Table 3.** Empowerment and Self-efficacy Post-Training

Variable	Post-training % Agreed (n)
**Empowerment & Self-efficacy**	
If one of my friends is using an e-cigarette or vaping product, I would feel comfortable encouraging them to quit.	87.50 (14)
There is something I can do to change the trend of using e-cigarette or vaping products in my school.	87.50 (14)
I am confident that I can select effective strategies to educate young people to lower tobacco use in my community.	87.50 (14)
In the future, I would be interested in forming a student group to support efforts to lower tobacco use in my community.	87.50 (14)
